# A Simple Fecal Bacterial Marker Panel for the Diagnosis of Crohn’s Disease

**DOI:** 10.3389/fmicb.2019.01306

**Published:** 2019-06-12

**Authors:** Songhe Guo, Yongfan Lu, Banglao Xu, Wan Wang, Jianhua Xu, Ge Zhang

**Affiliations:** ^1^Department of Microbial and Biochemical Pharmacy, School of Pharmaceutical Sciences, Sun Yat-sen University, Guangzhou, China; ^2^Department of Clinical Laboratory, Guangzhou First People’s Hospital, Guangzhou, China; ^3^Department of Laboratory Science, The Second Affiliated Hospital of Guangzhou University of Chinese Medicine, Guangzhou, China; ^4^Laboratory of Oncology Science and Molecular Biology, ShunDe Hospital of Guangzhou University of Chinese Medicine, Foshan, China

**Keywords:** *Fusobacterium nucleatum*, *Faecalibacterium prausnitzii*, Crohn’s disease, diagnosis, fecal bacteria

## Abstract

**Background and Aims:** Intestinal dysbiosis is implicated in the pathogenesis of Crohn’s disease (CD). We evaluated fecal and sera microbial markers for clinical use in detecting CD.

**Methods:** Fecal samples from 346 Asian subjects were collected, including 95 patients with CD, 81 patients with ulcerative colitis (UC), 65 patients with irritable bowel syndrome (IBS), and 105 healthy subjects (HS). Microbial indicators *Fusobacterium nucleatum* (*Fn*), *Faecalibacterium prausnitzii* (*Fp*), and *Escherichia coli* (*E. coli*) were identified based on a review of the literature. The relative abundance of the three bacterial markers were measured by qPCR, and two serological microbial markers (anti-*Fn*, anti-*E. coli*) were measured by ELISA. We evaluated the diagnostic performance of these microbial markers by ROC curve analysis.

**Results:** The quantification of *Fp*, *Fn*, and *E. coli* of fecal samples is relatively stable when stored up to 6 h at room temperature. The significant increasing abundances of *Fn* were accompanied by a decline of *Fp* in the CD group. *Fn* exhibited a slightly higher diagnostic value than *Fp* in distinguishing CD from HS (Area Under Curve, AUC = 0.841 *vs*. 0.811) or irritable bowel syndrome (IBS) groups (AUC = 0.767 *vs*. 0.658), and the further combination of *Fn* and *Fp* improved the diagnostic value (HS, AUC = 0.867; IBS, AUC = 0.771). However, anti-*E. coli* and *anti-Fn* antibodies in serum did not possess diagnostic value for CD or UC.

**Conclusion:** A combination of fecal *Fn* and *Fp* was identified as a valuable marker for CD diagnosis. A CD bacterial marker panel may provide a simple non-invasive approach to screen for CD.

## Introduction

The intestinal microbiome is a key factor in the development and maintenance of mucosal homeostasis (Sartor, 2015), and dysbiosis is closely involved in the pathogenesis of inflammatory bowel disease (IBD). In Crohn’s disease (CD), it is thought to play a role in initiating and triggering the immune system, leading to characteristic inflammation (Sokol et al., 2008; Joossens et al., 2011).

Several bacterial species have been implicated in CD by direct detection or by disease-associated antimicrobial immune responses. Many studies have started to investigate microbial features as potential biomarkers for CD (Manichanh et al., 2006; Jansson et al., 2009). The reduced presence of *Faecalibacterium prausnitzii* (*Fp*) has been well documented in patients with CD as opposed to controls (Willing et al., 2010; Swidsinski et al., 2011; Miquel et al., 2013; Pascal et al., 2017) and was further identified to be associated with a higher risk of CD recurrence (Sokol et al., 2008; Rajca et al., 2014). Interestingly, *Fusobacterium nucleatum* (*Fn*)-associated dysbiosis was found in gastrointestinal disease, including colorectal cancer (CRC) and CD (Yu T.C. et al., 2017; Liu et al., 2018), and highly invasive strains of *Fn* were suggested to be useful biomarkers for IBD (Strauss et al., 2011). More recent studies demonstrated that the fecal microbiota of CD is markedly increased in *Fusobacteriaceae* and *Escherichia* coupled with a decrease in *F. prausnitzii* (Gevers et al., 2014; Haberman et al., 2014). Together with our previous study, our data show that *Fn* has strong bactericidal activity against *Fp* (Guo et al., 2018), giving cause for optimism that assessing the “key microbial signature” may hold prognostic promise in CD.

Additionally, although the CD response to intestinal microbiota is yet to be discovered, these serologic microbial associated markers can be helpful to distinguish between CD and ulcerative colitis (UC), as well as aiding in the diagnosis of IBD (Bossuyt, 2006). The early available CD serological markers included antibodies to *Saccharomyces cerevisiae*, *Escherichia coli*, and *Pseudomonas fluorescens* (Targan et al., 2005; Olbjorn et al., 2017; Takedatsu et al., 2018). However, antibodies to obligate anaerobes, which are the predominant residents in the intestine, have not been investigated. Our recent study confirmed that *Fn* is a facultatively intracellular bacteria (Xue et al., 2018) that elicits a high level of serum anti-*Fn* antibodies in CRC patients (Wang et al., 2016). The use of a combination of serologic and fecal markers may additionally be incorporated into patient assessment.

In the present study, we detected the stool-based bacterial candidate markers in IBD patients and two control groups: irritable bowel syndrome (IBS) and healthy subjects (HS), and we detected the serologic microbial markers in CD and HS groups. Our study aimed to assess whether these bacterial markers were associated with clinicopathological parameters and might be diagnostic biomarkers for CD.

## Materials And Methods

### Patients, Definitions, and Clinical Phenotypes

The diagnosis of new onset IBD was prospectively established based on symptoms and preliminary examinations during the outpatient visit and then verified by standard clinical, radiological, histological, and endoscopic criteria after admission for CD with small intestinal lesions detected by radiological examination. The diagnosis of IBS patients was based on the Rome-III criteria: no alarming symptoms, normal colonoscopy and normal histology. Balloon-assisted enteroscopy was performed to clearly observe the affected intestine in IBS patients. As for healthy controls, all participants were free of symptoms and had a normal clinical examination and abdominal ultrasonography.

Consecutive patients with IBD (*n* = 176 or IBS (*n* = 65) were recruited prospectively from Guangzhou First Municipal People’s Hospital and Guangdong Provincial Hospital of Chinese Medicine between May 2015 and May 2018. Healthy controls (HS; *n* = 105) were recruited from the Medical Examination Center of Guangdong Provincial Hospital of Chinese Medicine. Inclusion criteria were: symptoms lasting for at least 3 months; complete colonoscopy with intubation of the terminal ileum, including biopsies; age of 13–77 years; informed consent; and fecal samples collected within 2 days before the colonoscopy (before bowel preparation). Use of antibiotics or drugs within the past 3 months was the exclusion criterion in this study. The patient characteristics are described in Table 1. Subjects were gender matched for all the groups. Concerning age, CD patients were younger than those in the HS group (*P* = 0.004).

**TABLE 1:** Sample size and clinical characteristics of subjects.

	**Healthy controls**	**IBD**		**Irritable bowel syndrome (IBS)**	***p***
**Baseline clinical characteristics**	**(*n*)**	**Crohn’s disease (*n*)**	**Ulcerative colitis (*n*)**	**(*n*)**	
***n* (patient)**	105	95	81	65	
**Gender**					0.74
Male	56	43	40	35	
Female	49	52	41	30	
**Age (years old)**					0.004
<16	0	13	7	1	
17–40	63	45	42	40	
>41	42	37	32	24	
**Active**					0.25
yes	na	69	57	na	
no	na	26	24	na	
**Location (Montreal classification)**					na
Ileal (L1)	na	38	na	na	
Colonic (L2)	na	15	na	na	
Ileocolonic (L3)	na	42	na	na	
**Behavior (Montreal classification)**					na
Non-stricturing, non-penetrating (B1)	na	9	na	na	
Stricturing (B2)	na	71	na	na	
Penetrating (B3)	na	15	na	na	
**UC classification**					na
Ulcerative proctitis (E1)	na	na	17	na	
Distal UC (E2)	na	na	40	na	
Extensive UC or pancolitis (E3)	na	na	24	na	
**IBS subtype**					na
Diarrhea predominant type	na	na	na	38	
Constipation predominant type	na	na	na	27	

### Blood and Stool Samples

Stool samples and corresponding sera samples were collected. A 2-ml fecal sample from each participant was collected and stored at room temperature for no more than 6 h; a 5-ml blood sample from each participant was allowed to clot for 30 to 60 min at room temperature. Each clotted sample was centrifuged at 4,000 rpm for 10 min. All sera and fecal samples were then aliquoted and frozen at −80°C until use.

To investigate the effects of storage conditions, eight fecal samples from healthy volunteers without inflammation were divided into aliquots and stored at room temperature. At the indicated time points, an aliquot of feces from each sample was moved to −80°C and the entire set underwent DNA extraction 1 month after the experiment began.

### Compliance With Ethical Standards

Ethical approvals were granted by the Ethics Committee of Guangzhou First Municipal People’s Hospital (No. K2016-001-01) and the Guangdong Provincial Hospital of Chinese Medicine (BF2018-055), with all methods carried out in accordance with the approved guidelines. Written informed consent was required for the enrollment of all patients into the study.

### Identified Microbial Candidates

Dates of publication for inclusion spanned from 2006 until 2017. Articles were collected through two psychology-focused journal databases: PubMed and Web of Science. Four groups of keywords were used interchangeably in separate searches of articles related to involvement and articles related to intervention. All possible combinations for the searches included one of the following keywords related to IBD: “*Fusobacterium*,” “*Faecalibacterium*,” “*Escherichia coli*,” or “Crohn’s disease.”

A search of the PubMed databases was performed using the keywords “Serologic,” “antibody,” “Crohn’s disease,” or “IBD.” The reference lists of the assessed articles were also searched for relevant studies.

### DNA Extraction and qPCR

Fecal DNA extraction and real-time quantitative PCR were performed as described previously. Briefly, total fecal DNA extraction was performed using the DNA Stool Mini Kit according to the manufacturer’s instructions (Tiangen, Beijing, China). The quantification of the target of the microbiomes and the reference gene (universal 16S rDNA) was performed on a LightCycler^®^480 II (Roche, Applied Science) using a SYBR green-based assay (Bio-Rad, United States). A positive/reference control and a negative control (H_2_O as template) were included within every experiment. Measurements were performed in triplicates for each sample. After the qPCR run, we performed melting curve analysis and gel electrophoresis to verify that the expected target was amplified. The primers used in the qPCR reaction are listed in Supplementary Table 1. Plasmid DNA containing the respective amplicon was diluted in 10-fold increments (10^8^–10^1^ copies) and used as quantification standards. Universal 16S rDNA was used as internal control and the abundances of gene biomarkers were expressed as relative levels to 16S rDNA (Yu J. et al., 2017).

### Bacterial Cultures

The *F. nucleatum* strain ATCC 25586, *F. prausnitzii* strain ATCC 27768, and *E. coli* strain ATCC 25922 were purchased from the Institute of Microbiology of Chinese Academy of Sciences. *Fn* and *Fp* were grown anaerobically at 37°C for 48 and 72 h, respectively, in a CDC anaerobic blood agar plate (Guangzhou Detgerm Microbiology Technology Co., Ltd., China) or 48 h in brain heart infusion (Oxoid, United Kingdom) broth culture before harvesting, while *E. coli* strains were cultured aerobically at 37°C for 24 h in an LB agar plate.

### ELISA

Serum-specific anti-bacteria antibodies were determined by an indirect whole-cell and extracellular protein ELISA, as described previously^22^. For whole-cell ELISA, 96-well plates were treated with 2.5% glutaric dialdehyde at 37°C for 2 h. Then, the heated-inactivated *Fn* and *E. coli* (1 × 10^8^ CFU/ml) were added and incubated at 37°C until the solution was dry. For extracellular protein ELISA, the extracellular proteins were obtained from bacterial supernatants. After being blocked with 1% BSA, the sample as incubated with a 2000-fold diluted human serum as a primary antibody and then with a goat anti-human IgG/IgA conjugated with HRP (Boster Biotechnology, China) as a secondary antibody. Blocking buffer was used for the determination of background values.

### Statistical Analysis

Values were all expressed as mean ± SD or median as appropriate. The differences in specific bacterial abundance were determined by Wilcoxon signed-rank test or Mann–Whitney *U* test. Continuous clinical and pathological variables were compared by *T*-test, whilst categorical variables were compared by Chi-square test. Pearson’s correlation coefficient was used to estimate the association of the bacterial abundances and several factors of interest. Factors independently associated with CD diagnosis were estimated using univariate and multivariate logistic regression. The performance of the markers was analyzed by calculating the area under the receiver-operating characteristic curve (AUC). Combination of *Fn* and *Fp* (*Fn-Fp*) was performed by fitting the markers into a binary logistic regression model, which used a logit function from binomial distribution to link the composite score and outcome. The *Fn/Fp* was calculated as log10(*Fn/Fp*). The best cutoff values were determined by ROC analyses that maximized the Youden index (J = Sensitivity + Specificity −1). The sensitivities, specificities, positive predictive value (PPV) and negative predictive value (NPV) were compared using the McNemar paired comparison test. All tests were done by Graphpad Prism 5.0 (Graphpad Software Inc., San Diego, CA, United States) or SPSS software v16.0 (SPSS, Chicago, IL, United States). *P* < 0.05 was considered statistically significant.

## Results

### Overview of Fecal Bacterial Marker *Fusobacterium*, *Escherichia coli*, and *Faecalibacterium* in Crohn’s Disease

We investigated the abundance of *Fusobacterium*, *Escherichia coli*, and *Faecalibacterium* in published CD data sets. There were 27 studies related to microbial features of CD that were included in the analysis, and the 909 CD and 768 HS samples were collected in China, America, and Europe from 2006 to 2017. Information about the selected studies is shown in Supplementary Table 2. These studies identified that *Fusobacterium*, *Escherichia coli*, and *Faecalibacterium* present opposite proportions in CD and HS groups.

Furthermore, our review of the literature identified data analysis from 4 studies published from 2001 to 2008 on CD microbial markers, indicating that IBD serological markers are useful in the diagnosis of IBD and the differentiation between CD and UC (Supplementary Table 3). No information about IBS anti-microbial antibodies was reported.

### Change in Fecal Bacterial Quantification During Collection and Storage

Whether the quantification of the target microbial markers is stable for fecal samples stored at room temperature is scarcely studied. We first examined the impact of different storage conditions on the stability of fecal microbial markers in eight healthy volunteers.

**FIGURE 1:**
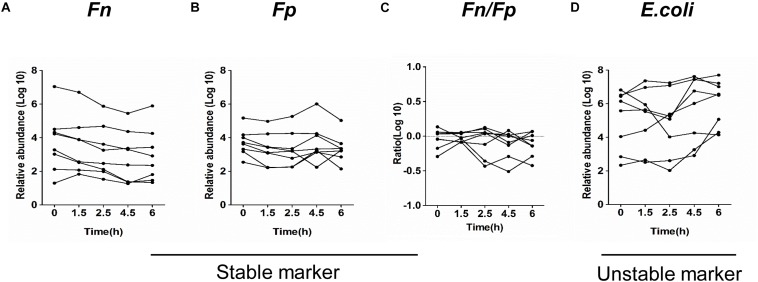
Quantitative detection of fecal bacterial markers in fecal samples in different storage conditions. The relative abundance of *Fn* **(A)**, *Fp* **(B)**, *Fn/Fp* **(C)**, and *E. coli* **(D)** in 8 fecal samples stored at room temperature for the indicated time intervals.

The relative abundance of *Fn*, *Fp*, and *E. coli* in the fecal samples, which were stored at room temperature at different time intervals from 0 to 6 h, were investigated by qPCR. Although there is a slight decrease in the mean abundance of strict anaerobes *Fn* and *Fp* after 1.5 h and a slight increase in the facultative anaerobe *E. coli* after 4.5 h, no significant differences were found among the mean abundance of three bacteria compared to the immediately frozen stool samples (Figures 1A–D). In addition, Supplementary Figures 1A–D shows that the quantification of *Fn* and *Fp* in each sample is stable at room temperature at different storage time intervals for 0, 1.5, 2.5, 4.5, and 6 h. The ratio of *Fn* to *Fp* (*Fn/Fp*) also displayed similar results. However, the quantification of *E. coli* obviously increased in a time-dependent manner during storage at room temperature in 4 samples (sample 1, 2, 4, 7). These findings indicated that the quantification of some anaerobic bacteria *Fp* and *Fn* of fecal samples is relatively stable, but the facultative anaerobe *E. coli* might have the potential to proliferate in some samples while at room temperature for 6 h.

### Evaluation of Fecal Microbial Markers in Patients With Crohn’s Disease

**FIGURE 2:**
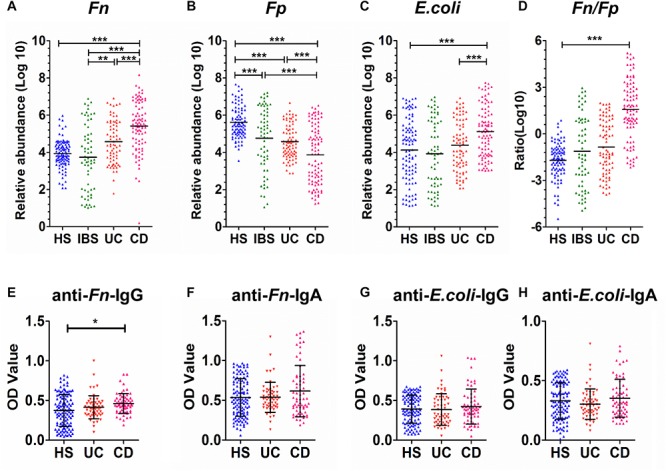
Quantitative detection of fecal and sera microbial markers in CD patients and control groups. **(A–D)** The relative fecal abundances of *F. nucleatum* (*Fn*) **(A)**, *F. prausnitzii* (*Fp*) **(B)**, and *E. coli* **(C)** ratio of *Fn* to *Fp* (*Fn/Fp*) **(D)** in 346 individuals, including 65 patients with IBS, 95 patients with Crohn’s disease (CD), 81 patients with ulcerative colitis (UC) and 105 healthy subjects (HS). **(E–H)** Comparison of OD values of anti-*Fn*/*E. coli*-IgG or anti-*Fn*/*E. coli-*IgA in sera from healthy subjects (HS, *n* = 105) and patients with ulcerative colitis (UC, *n* = 65) and Crohn’s disease (CD, *n* = 58) were individually assayed. Symbols indicate individual OD values; horizontal lines indicate mean values ± SD. Differences between the three groups were analyzed by the Kruskal–Wallis test. **(E)** Anti*-Fn*-IgG; **(F)** anti*-Fn*-IgA; **(G)** anti-*E. coli*-IgG; **(H)** anti-*E. coli*-IgA. ^*^P < 0.01, ^∗∗^*P* < 0.001, ^∗∗∗^*P* < 0.0001.

Furthermore, the carriage of *Fn*, *Fp*, and *E. coli* was investigated in fecal samples (*n* = 346) by qPCR. As shown in Figures 2A–C, the mean relative abundance of *Fn* was significantly higher, whereas the mean relative abundances of *Fp* were significantly lower in CD patients (*n* = 95) compared to patients with UC (*n* = 81), patients with IBS (*n* = 65) and healthy controls (*n* = 105; all *P* < 0.0001). However, the ratio of *Fn* to *Fp* (*Fn/Fp*) was only significantly higher in CD patients than in healthy controls (*P* < 0.01), but not in the IBS and UC groups (Figure 2D). Moreover, the mean relative abundance of *Fp* was significantly lower in UC than in the HS group, but there was no significant difference in *Fn* between the two groups.

Next, the mean relative abundance of *E. coli* was significantly higher in the CD group than in HS and UC groups (both *P* < 0.0001), but not in the IBS group. These results collectively suggested the potential of three bacterial marker candidates in discriminating CD patients. Particularly, the two strict anaerobes *Fn* and *Fp* showed good potential for differentiating CD from both HS and IBS controls.

### The Relationship Between the Microbial Indicators and the Clinicopathological Variables in Crohn’s Disease Patients

Associations between fecal microbial dysbiosis indicators and clinicopathological parameters and biochemical indexes in CD patients are presented in Table 2. The abundance of three fecal microbes, *Fp*, *Fn*, and *E. coli*, were not obviously correlated with UC age, gender, UC activation, UC classification (Supplementary Table 4).

**TABLE 2:** Relationship between the microbial indicators and the clinicopathological variables in Crohn’s disease (CD) patients.

		***Fn***		***Fp***		***E. coli***		***Fn/Fp***	
**Characteristics**	**n**	**(relative abundance) −(Log10)**	***p***	**(relative abundance) −(Log10)**	***P***	**(relative abundance) −(Log10)**	***p***	**(Log10)**	***p***
**CD**									
**Gender**			0.96		0.70		0.39		0.26
Male	43	2.50 ± 6.28		4.18 ± 6.51		3.01 ± 6.55		1.63 ± 2.45	
Female	52	2.56 ± 6.92		4.05 ± 6.41		2.78 ± 6.75		1.07 ± 2.33	
**Age (years old)**			0.04		0.98		0.01		0.87
<16	13	1.71 ± 6.41		4.05 ± 6.82		2.77 ± 6.34		1.49 ± 2.67	
17–40	45	2.85 ± 6.50		4.14 ± 6.45		3.30 ± 6.78		1.19 ± 2.44	
>41	37	2.80 ± 6.94		4.09 ± 6.34		2.43 ± 6.79		1.44 ± 2.29	
**Active**			0.01		0.30		0.06		0.22
yes	69	1.98 ± 6.97		4.38 ± 6.60		3.04 ± 6.59		1.50 ± 2.48	
no	26	2.78 ± 6.54		4.01 ± 6.42		2.46 ± 6.72		0.87 ± 2.12	
**Location (Montreal classification)**			0.98		0.41		0.09		0.47
Ileal (L1)	38	2.52 ± 6.44		4.10 ± 6.31		2.82 ± 6.51		1.42 ± 2.50	
Colonic (L2)	15	2.60 ± 6.40		3.78 ± 6.33		2.28 ± 6.80		0.64 ± 2.82	
Ileocolonic (L3)	42	2.57 ± 4.60		4.24 ± 6.65		3.16 ± 6.82		1.39 ± 2.09	
**Behavior (Montreal classification)**			0.40		0.34		0.19		0.94
Non-stricturing, non-penetrating (B1)	9	2.20 ± 6.71		4.69 ± 6.70		2.50 ± 6.88		1.29 ± 2.21	
Stricturing (B2)	71	2.51 ± 6.56		3.99 ± 6.48		2.84 ± 6.64		1.37 ± 2.40	
Penetrating (B3)	15	2.95 ± 6.75		4.36 ± 6.30		3.39 ± 6.73		1.12 ± 2.56	

*Median log10 bacterial copies/ratio ± standard deviations.*

However, there was a significant association between the presence of *Fn* and CD age, active (*P* = 0.04, *P* = 0.01). The abundance of *E. coli* was significantly associated with CD age (*P* = 0.01) (Table 2).

Furthermore, Pearson’s correlation coefficient and a linear regression analysis were applied to analyze the correlation between the *Fn*, *Fp*, and *E. coli* in HS, UC, CD, IBS patients, but there was no correlation between the three bacterial in the four group (Supplementary Figure 2).

### Diagnostic Value of the Microbial Indicators for Crohn’s Disease Patients

To determine whether those microbial indicators had diagnostic value for CD, the ROC curve was plotted to identify a cut-off value that would distinguish CD from HS (Supplementary Figure 3). As shown in Table 3, the marker *Fn* showed the best performance in discriminating CD from HS, with an area under the ROC curve (AUC) value of 0.841 (95% CI = 0.782–0.890), with a sensitivity of 73.63% and a specificity of 91.35%, a negative predictive value (NPV) of 88.2%, and a positive predictive value (PPV) of 79.8%; the marker *Fp* was 0.811 (95% CI = 0.748–0.864), with a sensitivity of 67.1% and a specificity of 89.25%, an NPV of 86.7%, and a PPV of 72.8%. The AUC value of the marker *E. coli* was only 0.665 (95% CI = 0.594–0.731), with a sensitivity of 91.58% and a specificity of 37.0%, an NPV of 58.0%, and a PPV of 82.0%.

**TABLE 3:** Performance of *Fn*, *Fp*, or *E. coli* alone and their combination for discriminating Crohn’s disease (CD) from healthy controls (HS) or irritable bowel syndrome (IBS).

	**Best cut-off**	**AUC**	**Sensitivity%**	**Specificity%**	**PPV%**	**NPV%**
**HS vs. CD**						
*Fn*	4.62	0.841 (0.782–0.890)	73.63	91.35	88.2	79.8
*Fp*	4.73	0.811 (0.748–0.864)	67.71	89.25	86.7	72.8
*E. coli*	3.97	0.665 (0.594–0.731)	91.58	37.00	58.0	82.2
*Fn/Fp*	0.27	0.782 (0.715–0.839)	70.33	81.72	78.8	73.1
*Fn-Fp*	0.51	0.867 (0.810–0.912)	80.78	90.00	93.3	80.7
**CD vs. UC**						
*Fn*	4.97	0.694 (0.617–0.764)	69.23	63.89	70.8	62.2
*Fp*	3.44	0.624 (0.551–0.698)	46.88	92.59	88.2	59.5
*E. coli*	3.65	0.644 (0.568–0.714)	61.05	62.96	65.9	58.0
*Fn/Fp*	0.50	0.729 (0.657–0.793)	75.00	67.90	73.5	69.6
*Fn-Fp*	0.30	0.733 (0.659–0.799)	79.12	63.89	73.5	70.8
**CD vs. IBS**						
*Fn*	5.01	0.767 (0.692–0.831)	68.13	76.19	80.5	62.3
*Fp*	4.82	0.658 (0.579–0.731)	69.79	57.14	71.3	55.4
*E. coil*	2.66	0.678 (0.600–0.750)	100	38.10	70.9	100
*Fn/Fp*	0.65	0.738 (0.662–0.801)	72.46	69.57	74.5	61.3
*Fn-Fp*	0.63	0.771 (0.698–0.834)	73.96	73.02	80.7	64.8
**IBD vs. IBS**						
*Fn*	3.69	0.707 (0.643–0.765)	83.54	53.38	82.0	55.0
*Fp*	4.82	0.616 (0.552–0.678)	67.80	57.27	81.5	38.3
*E. coil*	3.04	0.633 (0.568–0.769)	94.89	44.73	81.1	72.7
*Fn/Fp*	0.46	0.729 (0.660–0.785)	73.93	75.36	79.5	60.1
*Fn-Fp*	0.59	0.745 (0.683–0.800)	87.20	55.56	83.6	62.5

When differentiating CD from UC, the marker *Fn* had an AUC of 0.694 (95% CI = 0.617–0.764), at the best cutoff value, the marker *Fp* provided a sensitivity of 46.88% and a specificity of 92.59%; the marker *Fp* provided a sensitivity of 68.13% and a specificity of 76.19%, the AUC value of the marker *Fn* was 0.624 (95% CI = 0.551–0.698); the marker *E. coli* had an AUC of 0.644 (95% CI = 0.568–0.714) with a sensitivity of 61.05% and a specificity of 62.96% (Table 3).

The performance of *Fn* and *Fp* or *E. coli* in detecting CD from IBS. As shown in Table 3, the AUC of *Fn* reached 0.767 (95% CI = 0.692–0.831), respectively, whereas the AUCs for *Fp* and *E. coli* were only 0.658 and 0.678. These results suggested that the marker *Fn* and *Fp* (AUC = 0.841; 0.811), but not *E. coli* (AUC = 0.665), possessed good diagnostic capabilities for CD from HS.

### The Combination of *F. nucleatum* Improves the Diagnostic Ability of *F. prausnitzii* Alone for Crohn’s Disease Patients

Although both bacterial species were confirmed to be good indicators of CD diagnosis, we further investigated if the discriminatory power was enhanced when combining the markers *Fn* with *Fp* (*Fn*-*Fp*) for the diagnosis of CD. The performance of *Fn*-*Fp* in detecting CD from HS was assessed in 95 patients. As shown in Supplementary Figure 3E, the AUC of *Fn*-*Fp* reached 0.867 (95% CI = 0.809–0.912), whereas *Fp*, *Fn*, and *Fn/Fp* were 0.811, 0.841, and 0.782, respectively. At the best cutoff value, *Fn*-*Fp* offered a sensitivity of 94.62%, a specificity of 76.92%, an NPV of 93.3%, and a PPV of 80.7% (Table 3).

We found that a simple linear combination of *Fn* and *Fp* gave an increased AUC (0.733; 95% CI = 0.659–0.799) compared to *Fp* alone (0.624) and *Fn* alone (0.694). At the best cutoff value, *Fn*-*Fp* could discriminate CD from UC with a sensitivity of 79.12%, a specificity of 63.89%, an NPV of 73.5%, and a PPV of 70.8%, showing a better diagnostic performance than *Fp* or *Fn* only (Table 3). Nevertheless, for discriminating CD from IBS, the AUC of *Fn*-*Fp* increased slightly to 0.771 (95% CI = 0.698–0.834) compared to 0.658 for *Fp* and 0.767 for *Fn* alone (Table 3). However, the diagnostic value of *Fn-Fp* was limited for discriminate CD subtype from different intestinal disorders (HS: controls; IBS: Irritable Bowel Syndrome; E1: Ulcerative proctitis; E2: Distal UC; E3: Extensive UC), (all AUC <0.75, Supplementary Table 5).

These results suggested that the combination of bacterial markers had the highest sensitivity and specificity for the non-invasive diagnostic value of patients with CD.

### Evaluation of Serum *F. nucleatum* Antibodies in Patients With Crohn’s Disease

Furthermore, the anti-*Fn*, anti-*Fp*, and anti-*E. coli* levels were investigated in sera samples by indirect whole-cell ELISA. The coated bacteria whole cells served as antigens to react with the sera (1:2000 diluted) of patients and controls for detecting potential antibodies present in the sera. As shown in Figures 2E,F, the serum for anti-*Fn*-IgG levels in the CD group (*n* = 58) exhibited a significantly higher mean level than healthy control groups (*n* = 105, only *P* = 0.029), but not higher than the UC group (*n* = 65), whereas the anti-*Fn*-IgA levels in serum showed no obvious differences. In addition, the circulating levels of anti-*E. coli* IgG or IgA showed no significant differences between the CD and two control groups (Figures 2G,H). Similar results were found using the *Fn* extracellular proteins in the indirect ELISA. Additionally, no anti-*Fp* antibodies were detected with the 1000 or 2000 fold diluted serum samples in both HS and IBD groups. Those results suggested that both anti-*E. coli*, anti-*Fn*, and anti-*Fp* antibodies did not possess diagnostic value for CD or UC.

## Discussion

Bacterial diversity is different from proximal to distal locations of the gastrointestinal tract. CD can affect any of those areas, but most commonly attacks the distal ileum, which has a concentration of 10^7^–10^8^ bacteria ml^–1^ and usually contains bacteria similar to those found in the colon (Okamoto et al., 2000; Testa et al., 2003). *Fn*-associated dysbiosis in the areas of the colon and the rectum is involved in colorectal carcinogenesis (Kelly et al., 2018). Our previous study demonstrated that the fecal microbial ratio *Fn/Bifidobacterium* and *Fn/Fp* are useful non-invasive screen markers for early CRC, and *Fn* had an antagonistic effect against the probiotics *Fp*, *Bifidobacterium lactis* and *Lactobacillus rhamnosus* (Guo et al., 2018). Cancer of the small intestine is very uncommon, and *Fn*-associated dysbiosis in the areas of ileum is involved in CD (Ray and Dittel, 2015). CD-associated dysbiosis was previously characterized by a loss of *Fp* (Quevrain et al., 2016), which is one of the most abundant anaerobic bacteria in the human gut microbiota, with a proportion of approximately 5% of total bacteria in feces (Manichanh et al., 2006). Our results further suggested that *Fn* exhibited a relatively higher abundance while *Fp* was reduced in stool samples from CD patients compared to the loads observed in HS.

Additionally, our results showed that the abundances *Fp* and *Fn* can differ among intestinal disorders and IBD phenotypes of CD. These markers were selected from two bacteria that were among the most significantly associated and formed a co-occurrence network in the IBD microbiota (Quigley and Quera, 2006; Lopez-Siles et al., 2016). We observed consistent associations in the two markers and identified *Fp* as a key marker. This is consistent with increasing evidence of the functional role of the bacterium in IBD and extends the potential utility of this marker from patient prognosis to IBD diagnosis. We demonstrated that the combined relative abundance of *Fn* and *Fp* exhibited a good potential for differentiating CD from both HS and IBS, but not for differentiating CD subtype from other intestinal disorders, suggesting that more biomarkers need to be explored. Although we previously reported that the *Fn/Fp* ratio is a quantitative indicator of intestinal dysbiosis in CRC patients (Guo et al., 2018), a major drawback of the use of fecal samples to determine the intestinal microbial composition is the fact that the fecal microbiota represents only the end of the colon. Therefore, the direct *Fn/Fp* ratio for CD may be interfered by the microbial composition in the colon, and we could only improve the diagnostic value (AUC >0.85) by the combination of *Fn* and *Fp* in this study.

The role of enteric microflora in CD pathophysiology is highlighted by the presence of antibody reactivity to microbial antigens. Among CD serological markers, the yeast *Saccharomyces cerevisiae* (ASCA) antibodies have the highest diagnostic value and exhibit approximately 50–70% sensitivity and 80–90% specificity in detecting CD (Kuna, 2013). However, many of the published studies have been criticized because of their cross-sectional nature regarding anti-bacteria antibodies and using different coating antigen in different studies. In our study, we demonstrated that although the level of anti-*Fn* antibodies is slightly increased, the increase of anti-*Fn* antibodies has not shown a good diagnostic capacity to differentiate CD patients from healthy subjects. Additionally, our results showed that the levels of anti-*E. coli* antibody were not significantly different between CD and healthy controls.

Stool-based bacterial diagnosis needs fresh or immediately frozen stool samples, so it is a logistically challenging to collect and store samples. Especially, our stool samples came from different hospitals and the potential bias derived from store samples due to the different storage and transportation conditions. Several prior studies have reported no significant variation in the composition of fecal samples stored at room temperature up to 24 h (Tedjo et al., 2015), but other studies showed that the composition of samples stored at room temperature changed substantially. These changes principally represented increases in the relative abundance of *Bifidobacterium* and decreases in the phylum *Firmicutes*, including *Faecalibacterium* (Roesch et al., 2009; Choo et al., 2015). There have been inconsistent conclusions about the storage conditions of fecal samples. Sample storage conditions are important for the unbiased analysis of microbial communities in metagenomic studies. Specifically, for gut microbiota studies, stool specimens are often exposed to room temperature (RT) conditions prior to analysis. This could lead to variations in the quantitative assessment of bacterial communities. Our study showed that the quantification of *Fn* and *Fp* during storage up to 6 h at room temperature is similar to that in samples frozen immediately, suggesting that DNA quantification for obligate anaerobes of stool samples is relatively stable at room temperature for no more than 6 h.

## Conclusion

In conclusion, this study identified fecal *Fp* and *Fn* as useful biomarkers for detecting CD. As this study uses a retrospective case-control design, more work is necessary to evaluate the effectiveness of this marker in an average risk population of appropriate age, sex and demographics. Furthermore, this study has not evaluated the microbial markers in other colorectal diseases, such as colorectal carcinoma, which may affect the microbiota and, thus, the performance of the markers. Nevertheless, this relatively simple approach of adding a single microbial marker will enhance clinical applicability. This study takes the field one step closer to a non-invasive, potentially more accurate and affordable diagnostic procedure for IBD.

## Ethics Statement

Ethical approvals were granted by the Ethics Committee of Guangzhou First Municipal People’s Hospital (No. K2016-001-01) and the Guangdong Provincial Hospital of Chinese Medicine (BF2018-055), with all methods carried out in accordance with the approved guidelines.

## Author Contributions

GZ, SG, and JX contributed to the conception and design of the study. SG and WW contributed to data acquisition, data analysis and manuscript writing. BX, YL, and GZ participated in data analysis and manuscript writing. All authors read and approved the final manuscript.

## Conflict of Interest Statement

The authors declare that the research was conducted in the absence of any commercial or financial relationships that could be construed as a potential conflict of interest.
