# Supplementary material for: A Simple Fecal Bacterial Marker Panel for the Diagnosis of Crohn’s Disease
Source: Front Microbiol. 2019 Jun 12;10:1306. doi: 10.3389/fmicb.2019.01306 (PMC6581672; doi:10.3389/fmicb.2019.01306)
Supplement: Supplementary file 1 [file Table_1.docx]

**Supplementary Information**

**Supplementary Table 1.** Primers in this study.

| **Targets** |  | **Nucleotide sequence (5'-3')** | **Reference** |
| --- | --- | --- | --- |
| Internal control (16S rDNA) | Forward | CGTCAGCTCGTGYCGTGAG | [1] |
|  | Reverse | CGTCRTCCCCRCCTTCC |  |
| *Fusobacterium nucleatum* | Forward | TTCAATAAAAGTGGCAGGTCAAG | [1] |
|  | Reverse | TAACAACACATGCAGGTCAATGG |  |
| *Faecalibacterium prausnitzii* | Forward | GGAGGATTGACCCCTTCAGT | [2] |
|  | Reverse | CTGGTCCCGAAGAAACACAT |  |
| *Escherichia coli* | Forward | CATGCCGCGTGTATGAAGAA | [3] |
|  | Reverse | CGGGTAACGTCAATGAGCAAA |  |

**Reference**

1. Liang Q, Chiu J, Chen Y, Huang Y, Higashimori A, Fang J, et al. Fecal Bacteria act as novel biomarkers for non-invasive diagnosis of colorectal cancer. *Clin Cancer Res.* 2017;23(8): 2061-2070.
2. Kabeerdoss J, Sankaran V, Pugazhendhi S, Ramakrishna BS. Clostridium leptum group bacteria abundance and diversity in the fecal microbiota of patients with inflammatory bowel disease: A case-control study in india. *BMC Gastroenterol.* 2013;13(1):20.

Huijsdens XW, Linskens RK, Mak M, Meuwissen SG, Vandenbroucke-Grauls CM, Savelkoul PH. Quantification of bacteria adherent to gast rointestinal mucosa by real-time PCR. *J Clin Microbiol.* 2002;40(12): 4423-4427.

**Supplementary Table 2.** The abundance of *Fusobacterium, Escherichia coli* and *Faecalibacterium* in published Crohn’s disease (CD) data sets.

| **Technique** | ***Fusobacterium*** | ***Faecalibacterium*** | ***Escherichia coil*** | **n** | **Sample** | **Reference** |
| --- | --- | --- | --- | --- | --- | --- |
| qPCR | increase | - | - | 67 | feces | [1] |
| 16S rRNA sequencing | increase | reduce | - | 447 | feces | [2] |
| qPCR | - | reduce | - | 26 | feces | [3] |
| Pyrosequencing | - | reduce | - | 50 | feces | [4] |
| 16S rRNA sequencing | - | reduce | - | 20 | feces | [5] |
| 16S rRNA sequencing | - | reduce | - | 68 | feces | [6] |
| qPCR | - | reduce | - | 47 | feces | [7] |
| qPCR | - | reduce | - | 21 | mucosa | [8] |
| 16S rRNA sequencing | increase | reduce | - | 31 | mucosa | [9] |
| 16S rRNA sequencing | - | reduce | - | 36 | mucosa | [10] |
| qPCR | - | reduce | - | 45 | mucosa | [11] |
| 16S rRNA sequencing | - | reduce | - | 73 | feces | [12] |
| qPCR | - | reduce | - | 34 | feces | [13] |
| 16S rRNA sequencing | increase | reduce | - | 15 | feces | [14] |
| 16S rRNA sequencing | increase | reduce | - | 24 | feces | [15] |
| 16S rRNA sequencing | increase | reduce | - | 34 | feces | [16] |
| FISH | increase | reduce | - | 100 | feces | [17] |
| 16S rRNA sequencing | increase | reduce | - | 40 | feces | [18] |
| qPCR | increase | reduce | - | 67 | feces | [19] |
| qPCR | increase | reduce | - | 6 | feces | [20] |
| 16S rRNA sequencing | increase | reduce | - | 87 | feces | [21] |
| qPCR | - | reduce | increase | 45 | mucosa | [22] |
| 16S rRNA sequencing | - | reduce | increase | 6 | feces | [23] |
| qPCR | - | reduce | increase | 31 | feces | [24] |
| qPCR | - | - | increase | 40 | feces | [25] |
| 16S rRNA sequencing | - | reduce | increase | 40 | feces | [26] |

**Reference**

1. Andoh A, Kuzuoka H, Tsujikawa T, Nakamura S, Hirai F, Suzuki Y, et al. Multicenter analysis of fecal microbiota profiles in Japanese patients with Crohn’s disease. *J Gastroenterol*. 2012; 47(12): 1298-1307.
2. Kang S, Denman SE, Morrison M, Yu Z, Dore J, Leclerc M, McSweeney CS. Dysbiosis of fecal microbiota in Crohn's disease patients as revealed by a custom phylogenetic microarray. *Inflamm Bowel Dis*. 2010; 16(12): 2034-2042.
3. Quigley EM, Quera R. Small intestinal bacterial overgrowth: roles of antibiotics, prebiotics, and probiotics. *Gastroenterology.* 2006;130:S78-90.
4. Willing B, Halfvarson J, Dicksved J, Rosenquist M, Järnerot G, Engstrand L, et al. Twin studies reveal specific imbalances in the mucosa-associated microbiota of patients with ileal Crohn's disease. *Inflamm Bowel Dis.* 2009;15:653-60.
5. Conte MP, Schippa S, Zamboni I, Penta M, Chiarini F, Seganti L, et al. Gut-associated bacterial microbiota in paediatric patients with inflammatory bowel disease. *Gut*. 2006;55:1760-7.
6. Zhou Y, Chen H, He H, Du Y, Hu J, Li Y, et al. Increased *Enterococcus faecalis* infection is associated with clinically active Crohn disease. *Medicine*. 2016;95(39):e5019.
7. Gevers D, Kugathasan S, Denson LA, Vázquez-Baeza Y, Van Treuren W, Ren B, et al. The treatment-naive microbiome in new-onset Crohn’s disease. *Cell Host Microbe*. 2014; 12;15(3):382-392.
8. Lopez-Siles M, Martinez-Medina M, Busquets D, Sabat-Mir M, Duncan SH, Flint HJ, et al. Mucosa-associated *Faecalibacterium prausnitzii* and *Escherichia coli* co-abundance can distinguish Irritable Bowel Syndrome and Inflammatory Bowel Disease phenotypes. *Int J Med Microbiol*. 2014;304(3-4):464-75.
9. Schäffler H, Kaschitzki A, Alberts C, Bodammer P, Bannert K, Köller T, et al. Alterations in the mucosa-associated bacterial composition in Crohn’s disease: a pilot study. *Int J Colorectal Dis*. 2016;31(5):961-71.
10. Lopetuso LR, Petito V, Graziani C, Schiavoni E, Paroni Sterbini F, Poscia A, et al. Gut Microbiota in Health, Diverticular Disease, Irritable Bowel Syndrome, and Inflammatory Bowel Diseases: Time for Microbial Marker of Gastrointestinal Disorders. *Dig Dis*. 2018;36(1):56-65.
11. Tronstad RR, Kummen M, Holm K, von Volkmann HL, Anmarkrud JA, Høivik ML, et al. Guanylate Cyclase C Activation Shapes the Intestinal Microbiota in Patients with Familial Diarrhea and Increased Susceptibility for Crohn's Disease. *Inflamm Bowel Dis*. 2017 ;23(10):1752-1761.
12. Takahashi K, Nishida A, Fujimoto T, Fujii M, Shioya M, Imaeda H, et al. Reduced Abundance of Butyrate-Producing Bacteria Species in the Fecal Microbial Community in Crohn's Disease. *Digestion*. 2016;93(1):59-65.
13. Fujimoto T, Imaeda H, Takahashi K, Kasumi E, Bamba S, Fujiyama Y, et al. Decreased abundance of *Faecalibacterium prausnitzii* in the gut microbiota of Crohn's disease. *J Gastroenterol Hepatol*. 2013;28(4):613-9.
14. Sokol H, Pigneur B, Watterlot L, Lakhdari O, Bermúdez-Humarán LG, Gratadoux JJ, et al. *Faecalibacterium prausnitzii* is an anti-inflammatory commensal bacterium identified by gut microbiota analysis of Crohn disease patients. *Proc Natl Acad Sci U S A*. 2008;105(43):16731-6.
15. Naftali T, Reshef L, Kovacs A, Porat R, Amir I, Konikoff FM, et al. Distinct Microbiotas are Associated with Ileum-Restricted and Colon-Involving Crohn's Disease. *Inflamm Bowel Dis*. 2016;22(2):293-302.
16. Lopez-Siles M, Martinez-Medina M, Abellà C, Busquets D, Sabat-Mir M, Duncan SH, et al. Mucosa-associated *Faecalibacterium prausnitzii* phylotype richness is reduced in patients with inflammatory bowel disease. *Appl Environ Microbiol*. 2015;81(21):7582-92.
17. Wang W, Chen L, Zhou R, Wang X, Song L, Huang S, et al. Increased proportions of *Bifidobacterium* and the Lactobacillus group and loss of butyrate-producing bacteria in inflammatory bowel disease. *J Clin Microbiol*. 2014;52(2):398-406.
18. Gałecka M, Szachta P, Bartnicka A, Łykowska-Szuber L, Eder P, Schwiertz A. *Faecalibacterium prausnitzii* and Crohn's disease is there any connection? *Pol J Microbio*l. 2013;62(1):91-5.
19. Shaw KA, Bertha M, Hofmekler T, Chopra P, Vatanen T, Srivatsa A, et al. Dysbiosis, inflammation, and response to treatment: a longitudinal study of pediatric subjects with newly diagnosed inflammatory bowel disease. *Genome Med*. 2016;8(1):75.
20. Goyal A, Yeh A, Bush BR, Firek BA, Siebold LM, Rogers MB, et al. Safety, Clinical Response, and Microbiome Findings Following Fecal Microbiota Transplant in Children With Inflammatory Bowel Disease. *Inflamm Bowel Dis*. 2018;24(2):410-421.
21. Choo JM, Leong LE, Rogers GB. Sample storage conditions significantly influence faecal microbiome profiles. *Sci Rep.* 2015;5:16350.
22. Quigley EM, Quera R. Small intestinal bacterial overgrowth: roles of antibiotics, prebiotics, and probiotics. *Gastroenterology.* 2006;130(2 Suppl 1):S78-90.
23. Lopetuso LR, Petito V, Graziani C, Schiavoni E, Paroni Sterbini F, Poscia A, et al. Gut Microbiota in Health, Diverticular Disease, Irritable Bowel Syndrome, and Inflammatory Bowel Diseases: Time for Microbial Marker of Gastrointestinal Disorders. *Dig Dis*. 2018;36(1):56-65.
24. Tronstad RR, Kummen M, Holm K, von Volkmann HL, Anmarkrud JA, Høivik ML, et al. Guanylate Cyclase C Activation Shapes the Intestinal Microbiota in Patients with Familial Diarrhea and Increased Susceptibility for Crohn's Disease. *Inflamm Bowel Dis*. 2017 ;23(10):1752-1761.
25. Takahashi K, Nishida A, Fujimoto T, Fujii M, Shioya M, Imaeda H, I, et al. Reduced Abundance of Butyrate-Producing Bacteria Species in the Fecal Microbial Community in Crohn's Disease. *Digestion*. 2016;93(1):59-65.
26. Kuna AT. Serological markers of inflammatory bowel disease. *Biochem Med*. 2013(1);23:28-42.

**Supplementary Table 3**. Anti-bacteria antibodies of serological markers of IBD.

Anti-Cbiri-antibody to bacterial flagellin; Anti-OmpC-antibody to outer membrane porin C; Anti-I2-antibody to the *Pseudomonas* fluorescens-associated sequence; ASCA-Anti-*Saccharomyces* cerevisiae antibodies.

| **Diagnosis** | **Antibody** | **Sensitivity (%)** | **Specificity (%)** | **Reference** |
| --- | --- | --- | --- | --- |
| **CD** | anti-CBiri | - | - | [1] |
|  | anti-OmpC | 20-55 | 81-88 | [2] |
|  | Anti-I2 | 42 | 76 | [3] |
|  | ASCA+/pANCA- | 46-64 | 92-99 | [4] |

**Reference**

1. Markowitz J, Kugathasan S, Dubinsky M, Mei L, Crandall W, LeLeiko N, et al. Age of Diagnosis Influences Serologic Responses in Children with CrohnÂ s Disease: A Possible Clue to Etiology? *Inflamm Bowet Dis*. 2008; 15(5): 714-71.
2. Ferrante M, Henckaerts L, Joossens M, Pierik M, Joossens S, Dotan N, et al. New serological markers in inflammatory bowel disease are associated with complicated disease behaviour. *Gut.* 2007;56:1394-403.
3. Mow WS, Vasiliauskas EA, Lin YC, Fleshner PR, Papadakis KA, Taylor KD, et al. Association of antibody responses to microbial antigens and complications of small bowel Crohn’s disease. *Gastroenterology*. 2004; 126(2): 414-421.
4. Koutroubakis IE, Petinaki E, Mouzas IA, Vlachonikolis IG, Anagnostopoulou E, Castanas E, et al. Anti-saccharomyces cerevisiae mannan antibodies and antineutrophil cytoplasmic autoantibodies in Greek patients with inflammatory bowel disease. *Am J Gastroenterotogy*. 2001; 96(2): 449.

**Supplementary Table 4.** Relationship between the microbial indicators and the clinicopathological variables in Ulcerative Colitis (UC) patients.

Note: Median log10 bacterial copies/ ratio ± standard deviations.

|  |  | ***Fn*** | | ***Fp*** | | ***E.coli*** | |
| --- | --- | --- | --- | --- | --- | --- | --- |
| **Characteristics** | **n** | **(relative abundance)**  **- (Log10)** | ***p*** | **(relative abundance)**  **- (Log10)** | ***p*** | **(relative abundance)**  **- (Log10)** | ***p*** |
| **UC** |  |  |  |  |  |  |  |
| **Gender** |  |  | 0.23 |  | 0.11 |  | 0.88 |
| Male | 40 | 3.58±6.85 |  | 3.58±7.22 |  | 3.59±6.56 |  |
| Female | 41 | 4.25±6.82 |  | 3.27±7.01 |  | 3.62±6.70 |  |
| **Age (years old)** |  |  | 0.26 |  | 0.97 |  | 0.75 |
| ＜16 | 9 | 2.82±6.87 |  | 3.45±7.17 |  | 3.71±6.19 |  |
| 17-40 | 41 | 3.56±6.74 |  | 3.44±7.07 |  | 3.50±6.73 |  |
| ＞41 | 31 | 3.42±7.00 |  | 3.39±7.08 |  | 3.74±6.65 |  |
| **Active** |  |  | 0.99 |  | 0.67 |  | 0.68 |
| yes | 57 | 3.42±6.87 |  | 3.36±7.07 |  | 3.57±6.65 |  |
| no | 24 | 3.43±6.81 |  | 3.45±7.10 |  | 3.70±6.63 |  |
| **UC classification** |  |  | 0.54 |  | 0.84 |  | 0.15 |
| Ulcerative proctitis (E1) | 17 | 3.69±7.05 |  | 3.31±7.02 |  | 3.19±6.79 |  |
| Distal UC (E2) | 40 | 3.30±6.80 |  | 3.46±7.14 |  | 3.55±6.60 |  |
| Extensive UC or pancolitis (E3) | 24 | 3.42±7.72 |  | 3.44±7.06 |  | 4.00±6.69 |  |

**
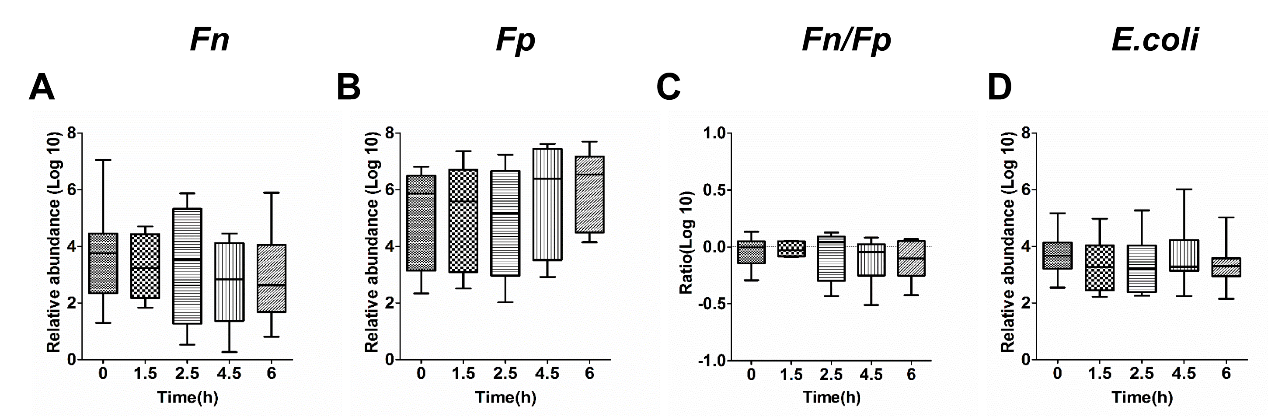
**

**Supplementary Figure 1. Quantitative detection of fecal bacterial markers in fecal samples in different storage conditions.**

The relative abundance of Fn **(A)**，*Fp* **(B)**，*Fn/Fp* **(C)** and *E. coli* **(D)** in 8 fecal samples stored at room temperature for the indicated time intervals. horizontal lines indicate mean values ± SD.

**
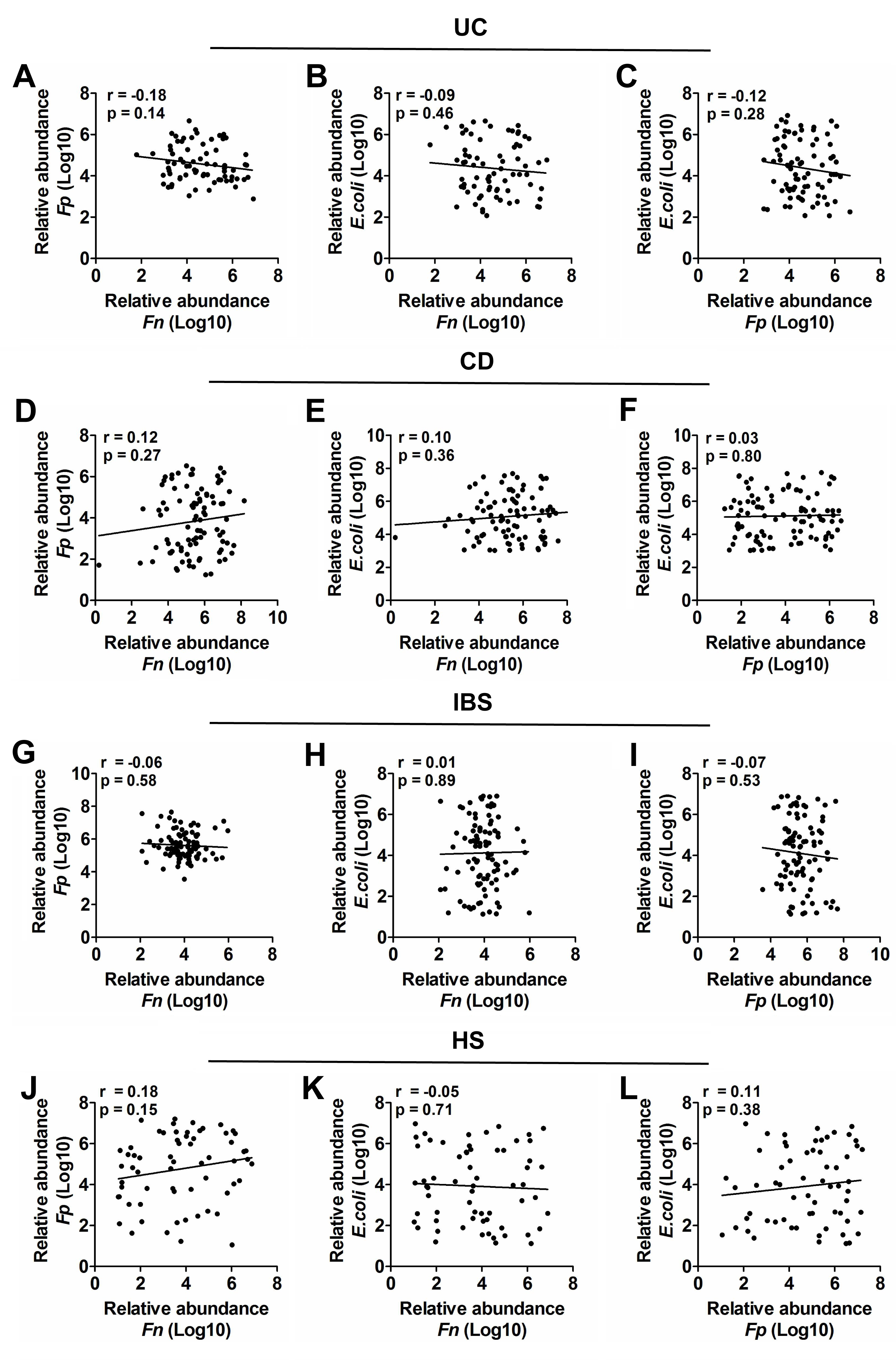
**

**Supplementary Figure 2. Correlation between fecal microbial markers in IBD,IBS patients and HS**. Correlation between the relative abundances of **(A)** *F. nucleatum* (*Fn*) and *F. prausnitzii* (*Fp*); **(B)** *Fn* and *E.coli*; and **(C)** *Fp* and *E.coli* in fecal samples of UC patients (n = 81). Correlation between the relative abundances of **(D)** *Fn* and *Fp*; **(E)** *Fn* and *E.coli*; and **(F)** *Fp* and *E.coli* in fecal samples of CD patients (n = 95). Correlation between the relative abundances of **(G)** *Fn* and *Fp*; **(H)** *Fn* and *E.coli*; and **(I)** *Fp* and *E.coli* in fecal samples of IBS patients (n = 65). Correlation between the relative abundances of **(J)** *Fn* and *Fp*; **(K)** *Fn* and *E.coli*; and **(L)** *Fp* and *E.coli* in fecal samples of HS (n =105).

**
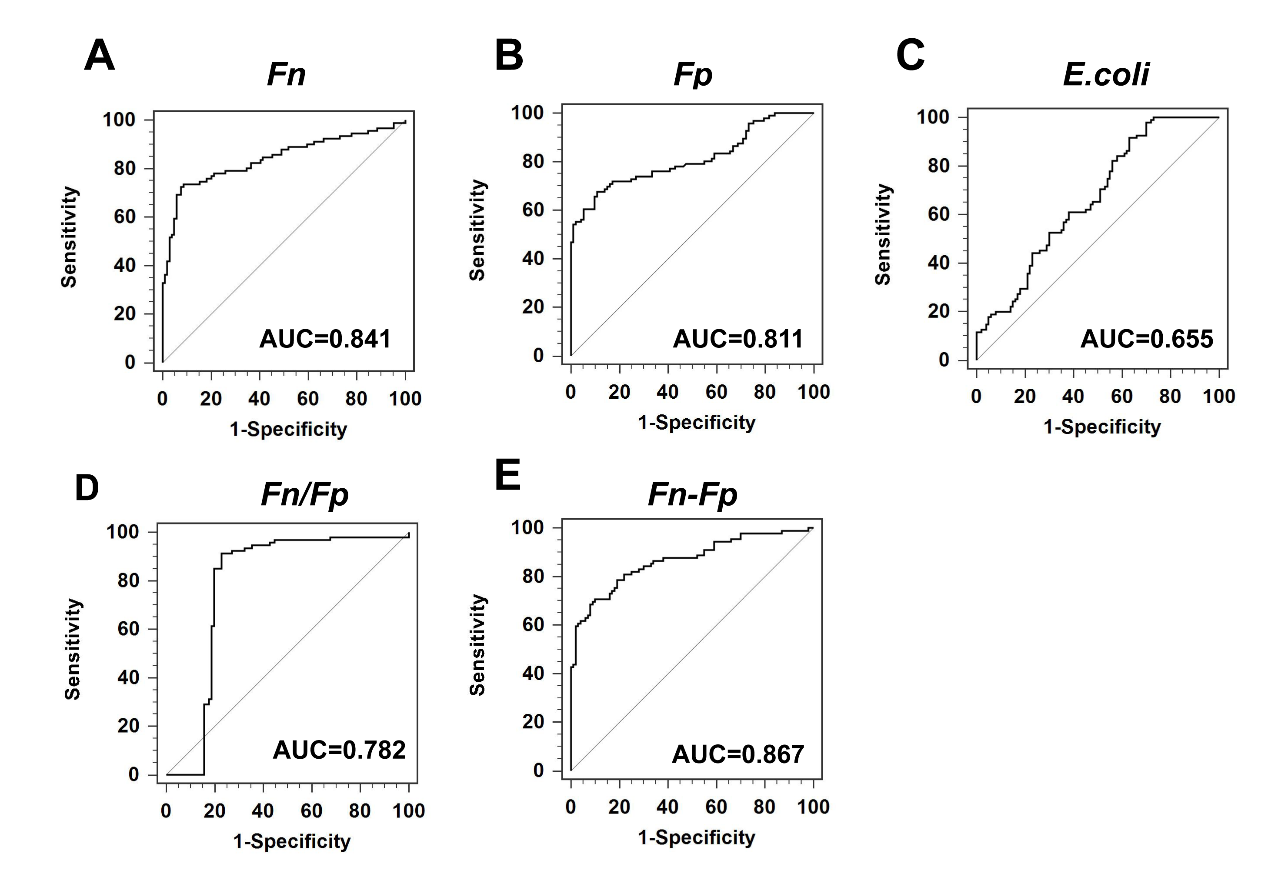
Supplementary Figure 3. Diagnostic outcomes for fecal microbial markers in the diagnosis of CD.**

ROC curves for *F. prausnitzii* (*Fp*) **(A)**, *F. nucleatum* (*Fn*) **(B)**, and *E. coli* **(C)**. AUC, area under the receiver-operating characteristic curve.

**Supplementary Table 5.** The combination of *Fn* and *Fp* (*Fn-Fp*) for discriminating amongst different intestinal disorders (HS, controls; IBS, Irritable Bowel Syndrome; E1, Ulcerative proctitis; E2, Distal UC; E3, Extensive UC or ulcerative pancolitis; I-CD, Ileal CD; IC-CD, Ileocolonic CD; C-CD, Colonic CD).

|  | **AUC** | **Sensitivity%** | **Specificity%** |
| --- | --- | --- | --- |
| ***Fn-Fp*** |  |  |  |
| I-CD vs HS | 0.716 | 77.14 | 66.77 |
| I-CD vs E1 | 0.655 | 74.29 | 60.00 |
| I-CD vs E2 | 0.698 | 60.00 | 78.24 |
| I-CD vs E3 | 0.619 | 54.29 | 75.83 |
| I-CD vs IBS | 0.611 | 38.57 | 80.95 |
|  |  |  |  |
| IC-CD vs HS | 0.698 | 75.00 | 67.85 |
| IC-CD vs E1 | 0.622 | 45.64 | 86.47 |
| IC-CD vs E2 | 0.681 | 65.26 | 77.50 |
| IC-CD vs E3 | 0.666 | 85.00 | 52.33 |
| IC-CD vs IBS | 0.573 | 48.31 | 68.25 |
|  |  |  |  |
| C-CD vs HS | 0.675 | 72.86 | 51.77 |
| C-CD vs E1 | 0.708 | 71.43 | 64.12 |
| C-CD vs E2 | 0.700 | 54.29 | 70.00 |
| C-CD vs E3 | 0.684 | 84.23 | 30.94 |
| C-CD vs IBS | 0.573 | 72.86 | 38.25 |
